# Female sex and burden of depressive symptoms predict insufficient response to telemedical treatment in adult attention-deficit/hyperactivity disorder: results from a naturalistic patient cohort during the COVID-19 pandemic

**DOI:** 10.3389/fpsyt.2023.1193898

**Published:** 2023-10-05

**Authors:** Peter Praus, Tanja Proctor, Tobias Rohrmann, Anastasia Benedyk, Heike Tost, Oliver Hennig, Andreas Meyer-Lindenberg, Anna-Sophia Wahl

**Affiliations:** ^1^Central Institute of Mental Health, University of Heidelberg, Mannheim, Germany; ^2^Institute of Medical Biometry (IMBI), University of Heidelberg, Heidelberg, Germany; ^3^Brain Research Institute, University of Zurich, Zurich, Switzerland; ^4^Department of Neuroanatomy, Institute of Anatomy, Ludwigs-Maximilians-University, Munich, Germany; ^5^Institute for Stroke and Dementia Research, University Hospital of Ludwigs-Maximilians-University, Munich, Germany

**Keywords:** telemedicine, attention-deficit/hyperactivity disorder, depression, WHO well-being, COVID-19

## Abstract

**Background:**

Attention-deficit/hyperactivity disorder (ADHD) is a chronic neuropsychiatric disorder, that typically manifests itself during childhood and persists in a majority of the affected individuals into adulthood, negatively affecting physical and mental health. Previous studies have shown detrimental effects of the COVID-19 pandemic on mental health in individuals with ADHD. Thus, telemedicine could be a useful tool for optimizing treatment-outcomes in adult ADHD by improving treatment adherence and persistence. However, data on telemedical treatment outcomes in adult patients with ADHD is scarce.

**Methods:**

We report here the sub-cohort analysis of a naturalistic cohort of adult patients (N = 254) recruited between April 2020–April 2021, comparing the effects of telemedical treatment on participants either clinically diagnosed with depression (N = 54) or ADHD (N = 67). Participants were asked to fill out the WHO-5 repetitively during >12 weeks of telemedical treatment. Furthermore scores of WHO-5, SCL-90R and BDI-II, psychopathology, psychosocial functioning, sociodemographic data, medical records and a feedback survey were analyzed for both groups and compared. Participants with ADHD were further stratified according to the development of well-being during the study period in order to identify factors associated with a satisfactory treatment outcome.

**Results:**

Participants with depression reported a significant improvement of well-being during the course of the study, while no such effect could be seen in participants with ADHD on a group level. Despite the good outcome, participants with depression were more severely affected at baseline, with significantly worse psychopathology and a more precarious labor and financial situation. A detailed analysis of ADHD participants without clinical improvement revealed significantly higher BDI-II scores than for ADHD participants with a satisfactory outcome (*p* = 0.03, Mann–Whitney-U-Test), suggesting successful treatment was hampered by the combination of ADHD and depressive symptoms. Furthermore, female sex among ADHD patients was correlated with an unfavorable treatment outcome during the course of the study (*p* = 0.001, Spearman correlation) as well as living with children (*p* = 0.02, Spearman correlation).

**Conclusion:**

Besides screening for depressive symptoms before telemedical treatment, future research should address the specific needs of female ADHD patients as these patients may be at a particularly high risk of being overburdened with family work.

## 1. Introduction

Attention-deficit/hyperactivity disorder (ADHD) is a common neuropsychiatric disorder comprising symptoms of inattention and hyperactivity-impulsivity, beginning during childhood and early adolescence and often persisting into adulthood (1, 2). Attention-deficit/hyperactivity disorder is highly heritable and of multifactorial etiology with environmental factors putatively contributing to the individual risk (3, 4). Prevalence estimates of ADHD in adulthood are flawed by methodological restrictions and the lack of large epidemiological studies of robust quality. In individual studies an estimated prevalence of, e.g., 4.4% could be demonstrated (5), whereas a meta-analysis and data from World Mental Health Surveys yielded a pooled prevalence of adult ADHD of 2.5% (6) and 2.8% (7) respectively.

The COVID-19 pandemic was associated with high rates of symptoms of insomnia, anxiety, depression and psychological distress in the general population (8), with younger age, female gender and the presence of chronic/psychiatric illnesses as prominent risk factors (9). In adolescents and young adults with ADHD the COVID-19 pandemic led to an exacerbation of psychosocial and mental health problems (10). Furthermore, there is compelling evidence that the pandemic, beyond co-occurring conditions, also exacerbated the core symptoms of ADHD (11, 12). In this line, there is evidence from a longitudinal Swiss cohort study that the prevalence of ADHD increased during the COVID-19 pandemic in a cohort of young adults, an effect that was exclusively seen among women (13). Additionally, adult individuals with ADHD in the US had a greater probability of hospitalization due to COVID-19. Comorbid substance use disorders significantly potentiated the risk of severe COVID-19 (14). Beyond that, perceived stress increased the risk for suicidal ideation in individuals with ADHD symptoms in an adult Japanese sample (15).

At the initial stages of the COVID-19 pandemic, telemedicine was - despite limited evidence - considered as an additional means of expanding the delivery of mental health services to patients with ADHD (16) and facilitating the remote monitoring of ADHD symptoms (17). Current findings, mainly relying on self-reports, suggest that digital interventions could improve medication adherence in children and adolescents with ADHD (18). Yet, the evidence remains inconclusive, due to risks of bias, heterogeneity of interventions and study designs, and low statistical power. In general, remote consultations via video, phone, or live-messaging are well accepted among people with mental health conditions (19), improving service quality from a patient perspective among adolescents and young adults (20). Likewise, videoconferencing seems to be an accessible and effective means of delivering behavioral and cognitive therapies to adults with mental health problems, e.g., post-traumatic stress disorder (PTSD), depression, anxiety disorders, eating disorders, and obsessive-compulsive disorder (OCD) (21). A recent systematic review (22) concluded that telemedicine had the potential to increase treatment availability, decrease diagnosis waiting times, and aid in symptom monitoring in patients with neurodevelopmental disorders (NDD), e.g., ADHD. Nevertheless, there is a preponderance of evidence for children and adolescents with NDD and their caregivers. In contrast, the evaluation of telemedical interventions for adult individuals with NDD is still underrepresented. Despite this nonetheless promising data situation, the response to the COVID-19 pandemic worsened problems with service provision to patients with ADHD in the United Kingdom, increasing the risk for negative long-term outcomes in these individuals (23). Meanwhile, data from Germany indicate an increase in pandemic-related symptoms of depression and anxiety and a decline of mental health in the general population during later stages of the pandemic (24). Even the rapid, yet initially somewhat uncoordinated widespread provision of telemedical psychiatric counseling and treatment options could not prevent this development. Correspondingly, we lately demonstrated an insufficient response of adult patients with ADHD to telemedical psychiatric treatment on a group level, compared to patients clinically diagnosed with a depressive disorder in a naturalistic, monocentric, exploratory trial during the first year of the COVID-19 pandemic in Germany (25). Therefore, in the light of the current shortcomings of evidence regarding the telemedical treatment of adults with ADHD, a better understanding of the determinants of telemedical treatment outcomes in this chronically affected group of patients is urgently needed.

The current analysis - building on the sample of the exploratory study mentioned above (25) – investigates clinically relevant characteristics of patients with ADHD, the group with the least favorable outcome during telemedical treatment in our study, by comparison with patients diagnosed with a depressive disorder, who reported the best response to telemedical treatment. Furthermore, patients’ subjective experiences with telemedical psychiatric treatment are compared.

## 2. Methods

### 2.1. Study design

Here, we report the analysis of a sub-cohort of our recently published study (25). This monocentric study was initiated during the first enforced Germany-wide lockdown (starting from March 22, 2020) due to the COVID-19 pandemic, when most services of the outpatient clinic at the Central Institute of Mental Health, Mannheim (CIMH), University of Heidelberg, Germany, had to be transformed into telemedical treatment options in order to maintain psychiatric services for patients with mental health issues despite severe contact restrictions. The study aimed at World Health Organization (1) observing how psychiatric symptoms of patients with mental health problems develop during the course of telemedical psychiatric treatment, American Psychiatric Association (2) identifying patient groups with favorable or less beneficial treatment outcomes, Thapar and Cooper (3) determining sociodemographic or related factors (sex, age etc.) with an impact on the effectiveness of telepsychiatric treatment, and Kim et al. (4) describing patients’ experiences with telepsychiatric consultations compared to conventional face to face treatment by mental health experts. Due to organizational reasons within the institution, patients seeking appointments at the department of addictive behavior and addiction medicine, the memory clinic, and the department of psychosomatic medicine at the CIMH were not systematically asked for their participation and therefore excluded. Thus, the study sample was restricted to adult, non-geriatric general psychiatric patients.

Recently, we determined a robust and favorable response to telemedical treatment among patients with depressive disorders in this patient cohort, whereas patients with chronic neurodevelopmental disorders like ADHD did not experience significant improvement at a group level (25). Therefore, we analyzed these two sub-cohorts of patients, either clinically diagnosed with ADHD (N = 67) or depression (N = 54), in order to identify relevant factors that are associated with divergent treatment outcomes. N = 22 participants diagnosed with ADHD had a depressive syndrome as secondary diagnosis and N = 2 of the patients diagnosed with ADHD had previously been diagnosed with a depressive episode. N = 5 patients diagnosed with depression had a reported secondary diagnosis of ADHD in the past documented in their medical records. All patients (N = 254) were recruited between April 2020 and April 2021. After their request for a first medical consultation, patients received information about the study during the telephonic scheduling of their first psychiatric counseling. The latter were exclusively offered via telemedicine (all 254 patients recruited) due to pandemic-related restrictions. The study was mainly observational. Thus, only children and adolescents (patients under 18 years of age) were excluded. Due to the naturalistic and observational nature of the study, no other exclusion criteria were applied. The aims and purpose of the study were explained either by members of the study team who contacted interested patients or by the psychiatrist or psychologist who provided the first telemedical session. Telemedical treatment was administered via phone or video calls, according to patients’ preferences, technical equipment as well as individual and legal data safety concerns. For initial telemedical appointments past and medical history of patients including current medical complaints as well as a history of psychiatric symptoms, treatments, medication, secondary diagnoses and social history were recorded comparable to an initial appointment in person except for the physical examination. Signs of psychopathology were also rated according to the AMDP system (26). During the following telemedical consultations, patients received psychiatric counseling with optimization of psychopharmacological treatment and/or psychotherapy. Between telemedical psychiatric consultations patients received scheduled appointments with strictly limited personal contact for blood tests, therapeutic drug monitoring (TDM), and physical as well as radiological examinations (e.g., MRI scans), if required. Participants were asked to complete three surveys during the course of the study: Before the first telemedical consultation, participants agreed that their medical record, which would be created during telemedical treatment, could be used for further analysis (see below) as a part of the study. Participants were also asked to fill out the WHO-5 well-being index (WHO-5) (27) and the symptom check-list-90-R (SCL-90R) (28), which were provided paper-based 4–6 and 8–12 weeks after the first telemedical session. Participants could either choose to take part in the second and third survey, according to their preference, via phone interview or by using online surveys on REDCap,^1^ a secure web application for building and managing online surveys for research studies and operations supported by the National Institutes of Health (NIH/NCATS UL1 TR000445). During all three surveys patients were asked to score their well-being according to the WHO-5 Well Being Index (see below). Patients using the online survey system REDCap were also asked to complete Beck’s depression inventory II (BDI-II) (29) during the second or third survey. At the end of the telemedical treatment patients could evaluate the psychiatric intervention either paper-based or via REDCap (see details below).

### 2.2. Acquisition of psychiatric history and sociodemographic data

All participants analyzed in this study (N = 67 with ADHD and N = 54 with depression) gave informed consent to analyze their medical records, including sex, age and sociodemographic data as well as their psychiatric history and standardized professional ratings of psychopathology for the purpose of the study. As described previously (25), all mental health experts providing telepsychiatric services in our outpatient clinic used a highly structured computerized rating of psychopathology provided by the electronic documentation system of our clinic (ORBIS, SAP, Walldorf Germany) during the first interview. Patients were screened for current psychiatric symptoms, psychiatric diagnoses according to the ICD-classification of the WHO (version 10), past psychiatric history and sociodemographic data, such as current living situation, education, professional training and labor situation, debts and history of criminal convictions. The demographic characteristics of the study sample are shown in Table 1. All psychiatrists and psychologists of our outpatient clinic were also requested to score patients according to the global assessment of functioning scale (GAF) and the clinical global impression scale (CGI) at baseline. During subsequent data analysis, electronic medical records were systematically queried for the number of telemedical treatment sessions participants received and possible hospitalizations during the course of the study. Medical records were additionally scrutinized for possible outpatient treatments during the year before March 2020, when outpatient psychiatric care was still provided personally.

**Table 1:** Sociodemographic characteristics of the participants diagnosed with depression or attention-deficit/hyperactivity disorder (ADHD) assessing sex, age, marital status, children, living situation, education, professional training, labor and financial situation.

	Depression		ADHD		
	N or M (SD)	(%)	N or M (SD)	(%)	*p*
Total Number (N) of recruited patients	54		67		
Female	39	72	36	58	**0.038**
Male	15	28	31	46	
Age (years)					
Females	37.3 (13.0)		40.8 (12.7)		0.861
Males	41.6 (10.4)		37.8 (12.4)		>0.999
Marital status					
Single	21	46	24	42	0.730
Living with a partner	25	54	33	58	
Missing responses	8		10		
Living situation					
Living alone	20	59	16	46	0.424
Living with family or friends	8	24	16	41	
Living with a partner	4	12	3	8	
Living in supervised accommodation	2	6	2	5	
Missing responses	20		28		
Education					
No school graduation	2	6	0	0	0.529
9 years of school education completed	6	17	10	18	
10 years of school education completed	12	33	17	31	
>12 years of school education completed	15	42	27	49	
Education not specified	1	3	1	2	
Missing responses	18		12		
Professional training					
Completed apprenticeship	24	73	25	51	0.121
Completed academic studies	2	6	12	24	
No completed professional training	4	12	6	12	
Academic studies on-going	3	9	6	12	
Missing responses	21		18		
Children					
Children	2	4	11	17	**0.037**
No children	18	38	29	45	
Children not specified	28	58	25	38	
Missing responses	6		2		
Labor situation					
Unemployed	10	21	9	14	**0.006**
Employed	11	23	30	46	
Disables	10	21	2	3	
Retired	1	2	3	5	
Labor situation not specified	16	33	21	32	
Missing responses	6		2		
Financial situation					
Debts	15	31	7	11	**0.015**
No debts	10	21	24	38	
Financial situation not specified	23	48	33	52	
Missing responses	6		3		

N = number of participants for whom information was found in medical records. Percentages were calculated as (N/all N responded for a distinct category)*100. Results are presented in mean ± SD for age; a Chi-squared test was used to compare patients with depression and patients with ADHD for different sociodemographic characteristics. *p* < 0.05 was set to be significant. Significant differences between groups for a specific category are marked in bold. M = mean, SD = standard deviation.

### 2.3. Evaluation and follow-up of psychopathological symptoms

#### 2.3.1. WHO-5 well-being index (WHO-5)

We used the WHO-5 for the assessment of overall well-being during telepsychiatric counseling. The WHO-5 is a short self-administered measure of well-being, covering the last 2 weeks before completion of the questionnaire (30). The WHO-5 consists of five positively worded items that are rated on a 6-point Likert scale, ranging from 0 (at no the time) to 5 (all of the time). We transformed the raw scores to a score from 0 to 100 (raw data*4), where lower scores indicated worse well-being. A score of ≤50 was considered as poor wellbeing and a score of 28 or below as indicative of depression. The WHO-5 has high clinimetric validity and can be used as an outcome measure for treatments. Moreover, the WHO-5 has proven its applicability across a wide range of study fields and is a valid screening tool for depression. For a comprehensive review of the psychometric properties and diagnostic accuracy of the WHO-5 see also Topp et al. (31).

#### 2.3.2. Symptom checklist-90-R

The SCL-90-R by Derogatis (32) measures the subjective perception of physical and mental symptoms a person has experienced during the past 7 days. All 90 symptoms are scored on a Likert scale consisting of 5 steps, ranging from 0 (no symptom at all) to 4 (very strong impairment due to the symptom). We analyzed the data according to the instructions provided by Derogatis and Franke for the German Version of the SCL-90-R (2nd Edition, Beltz Test, 2000). T values equal to and above 60 were considered as a relevant deviation from the respective symptom or global standard scores. The German version of the SCL-90-R has been validated in psychosomatic outpatients and primary care patients (33, 34). Despite the strong interdependence of its subscales the results of these studies indicated that the SCL-90-R is a useful tool for screening for mental disorders as well as measuring psychological status and change in outcome studies. However, due to a lack of factorial validity, the availability of representative norms for the German population are restricted to the global scores of the SCL-90-R (35). These three global indices (GSI: Global Severity Index, PST: Positive Symptom Total, PSDI: Positive Symptom Distress Index) provide measures of overall psychological distress by focusing on general psychological distress (GSI), the intensity (PSDI) and the number (PST) of symptoms.

#### 2.3.3. Beck’s depression inventory-II

During the second or third online survey via REDCap participants could also complete the BDI-II. 19 (35.2%) of the participants diagnosed with depression and 33 (49.3%) of the participants diagnosed with ADHD completed the BDI-II. The BDI-II is a widely used 21-item instrument, validated for the self-report of depressive symptoms experienced during the past 2 weeks (29, 36). Individual item scores (0 to 3) sum up to a total BDI-II score ranging from 0 to 63. BDI-II scores <13 were interpreted as indicative of minimal depressive symptoms without clinical relevance. Higher scores suggested a mild (14–19), moderate (20–28) or severe (29–63) burden of depressive symptoms. The German version of the BDI-II has recently been shown to be a reliable and valid screening tool for depressive disorders and episodes in the adult German population with high internal consistency (37).

#### 2.3.4. Clinical global impression scale (CGI) and global assessment of functioning scale (GAF)

While the WHO-5, SCL-90-R and the BDI-II are self-report questionnaires focusing on the subjective perception of overall well-being and different symptom domains, the GAF and CGI were used as clinician-rated scales to document the global impairment due to patients’ (mental) health conditions. CGI (38) scores indicate the severity of symptoms, ranging from 1 to 7 (1 = normal/not at all affected; 7 = very severely ill). The GAF (39) indicates the global functioning of a patient taking into account the psychiatric, social and professional level of functioning. The scale ranges from 0 (very sick) to 100 (healthy).

#### 2.3.5. Evaluation of telemedical treatment by participants

Thirty-two (59.3%) of the participants with depression and 45 (67.2%) of the participants with ADHD completed an evaluation questionnaire asking for feedback concerning technical details (e.g., if patients chose phone calls or video conferences or both, and if interruptions occurred due to technical problems). Moreover, patients were asked for their overall satisfaction with telepsychiatric consultations. Participants were asked how helpful they found the telemedical interventions during the study period and if they were comparable to conventional face to face consultations. Patients were also requested to state their preference about using telepsychiatry in the future again. Participants were also asked if and how the COVID-19 Pandemic had influenced their mental well-being.

### 2.4. Data analysis

All data acquired during surveys and from medical records were entered into an Excel master file and then translated to SPSS Version 27 and R Version 4.2.0.1. for further analysis. The descriptive statistics of the sample were computed for the sociodemographic characteristics, consisting of frequencies and percentages for categorical values and mean and standard deviations (SD) and for scale variables. Differences between two groups of patients (patients with depression vs. ADHD patients or ADHD patients with or without improvement on the WHO-5 well-being index) were assessed using the Mann-Witney-U test for the different non-parametric clinical scale variables or the Chi-Square tests for frequencies of the categories in the sociodemographic parameters.

**Figure 1:**
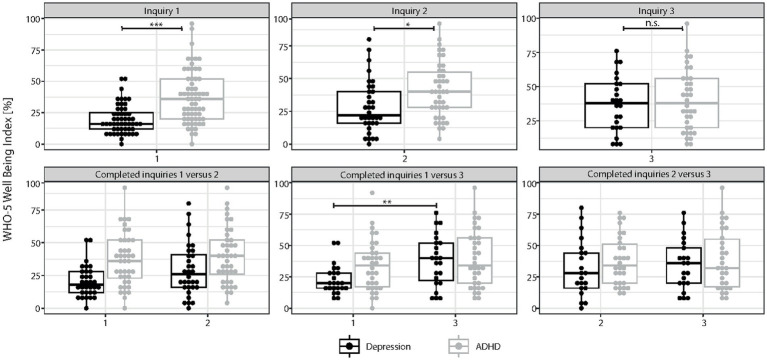
Results of self-report assessment using WHO-5. Results of the WHO-5 well-being index: Boxplots with scoring details of all individuals who participated in the different surveys of the study (inquiry 1 = survey before the beginning of telemedical treatment; inquiry 2 and 3 = surveys 4–6 and 8–12 weeks after the start of telemedical counseling, respectively) are shown for patients with depression and attention-deficit/hyperactivity disorder (ADHD). In the upper panel, all responses of patients, either diagnosed with ADHD or depression, are indicated for each of the three inquiry time points. In the lower panel, only results of participants who completed either inquiry 1 and 2 (N = 32 participants with depression and N = 40 participants with ADHD), inquiry 1 and 3 (N = 23 participants with depression and N = 34 participants with ADHD) or inquiry 2 and 3 (N = 21 participants with depression and N = 30 participants with ADHD) are shown. Statistical analyses could only be performed if the results of at least two subsequent inquiries could be compared. We found a significant improvement of WHO-5 scores during the course of telemedical treatment for participants diagnosed with depression (inquiry 1 vs. 2 and inquiry 1 vs. 3, *p* < 0.001, Wilcoxon-test) while this was not the case for participants diagnosed with ADHD. Results of individual study subjects are shown in percentages (0% indicates extremely impaired well-being while 100% represents perfect well-being). Results are presented in mean ± SD; asterisks indicate significances: *p < 0.05, **p < 0.01, ***p < 0.001.

**Figure 2:**
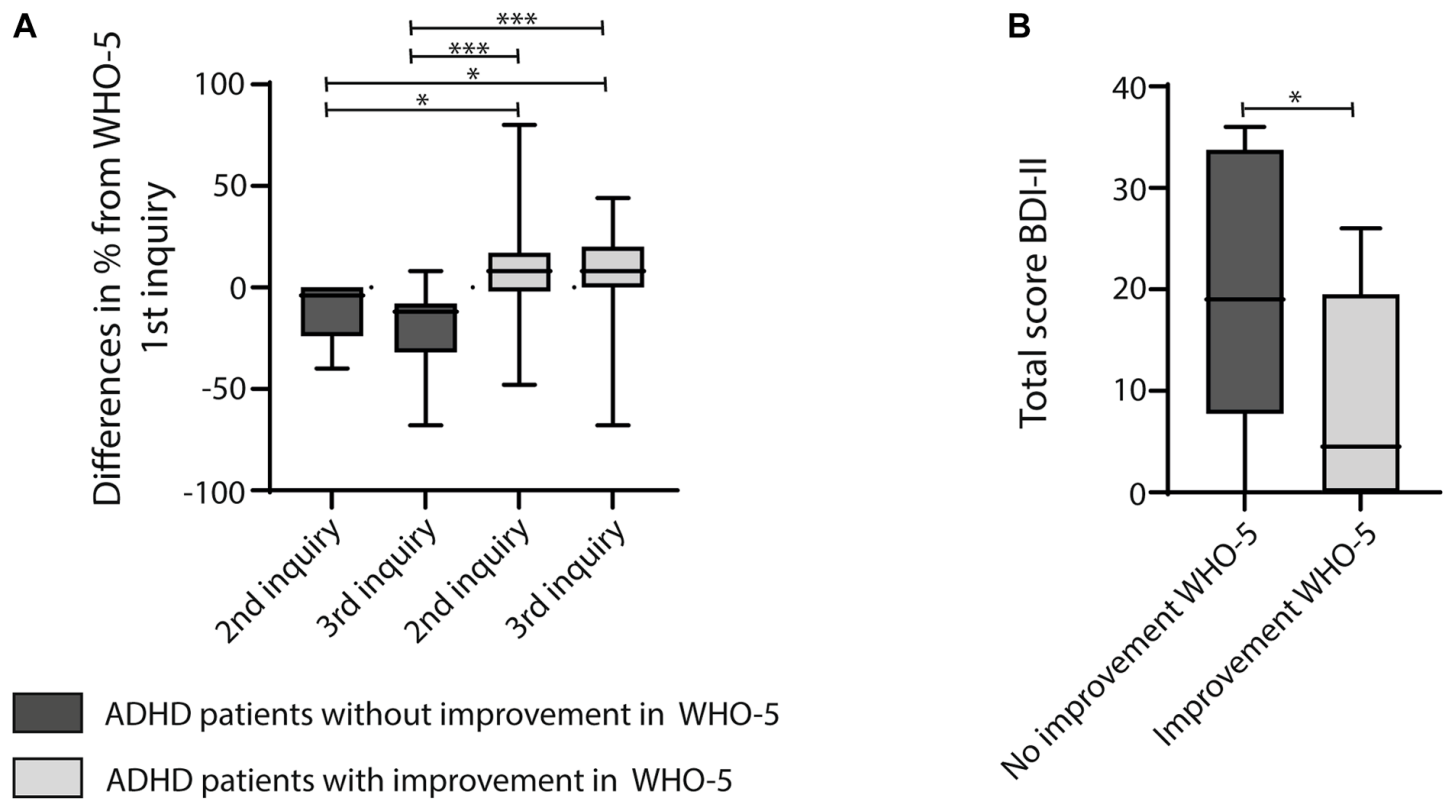
**(A)** Graph showing boxplots calculating differences in percentages of the WHO-5 scores between the 1st and 2nd inquiry or the 1st and 3rd inquiry of participants diagnosed with ADHD. The graph reveals one sub-cohort of participants with no improvement or even a decline in well-being on the WHO-5 during telemedical treatment, while another other sub-cohort of participants with ADHD reported enhanced well-being. The latter was significantly different from the sub-cohort without improvement. For statistical analysis the Kruskal-Wallis Test, multiple comparison with a Tukey *post-hoc* was used. **(B)** Participants with no improvement on the WHO-5 scored significantly higher on the BDI-II than participants who reported an improvement of well-being on the WHO-5 during the course of the telemedical intervention. Results were compared using the Mann–Whitney-U-Test; asterisks indicate significances: *p < 0.05, **p < 0.01, ***p < 0.001.

A paired Wilcoxon signed-rank test was used to assess differences in the levels of mental health variables, e.g., the results of the WHO-5 during subsequent inquiries. To this end, only data from subjects was included, who had participated in all inquiries (“complete cases;” Figures 1, 2). Correlations between variables (e.g., sex and outcome on the WHO-5) were assessed via Spearman’s correlation coefficient *r.* For all tests, a value of *p* of less than 0.05 was considered statistically significant.

### 2.5. Ethics approval and consent to participate

Written informed consent was obtained from all participants. The study design and data acquisition were presented to the ethics committee II of the medical faculty Mannheim, University of Heidelberg and approved (No. 2020-562 N).

## 3. Results

We previously reported that telemedical treatment in a naturalistic patient cohort (N = 254) during the COVID-19 pandemic supported mental well-being as measured by the WHO-5 well-being index and the SCL-90-R (25). The largest group of this patient cohort (35.3%) consisted of patients with neurodevelopmental disorders like ADHD and tic disorders, while almost a quarter of the study population (24.6%) was primarily diagnosed with a depressive episode or recurrent depressive disorder according to the ICD-10 classification. We now present sub-cohort analyses of these two patient cohorts, either diagnosed with depression (N = 54) or ADHD (N = 67). Wherever informed consent was given, sex, age and sociodemographic data as well as participants’ psychiatric history and standardized professional ratings of psychopathology from medical records were additionally evaluated.

### 3.1. Comparisons between patients with depression and patients diagnosed with attention-deficit/hyperactivity disorder

#### 3.1.1. Well-being, sociodemographic and sex-specific characteristics

Although participants with depression scored significantly lower on the WHO-5 than participants with ADHD at baseline (WHO-5 mean 20 ± 12 for participants with depression vs. mean 37 ± 21 for participants with ADHD, *p* < 0.001, Mann–Whitney-U-Test; Figure 1), depressive participants reported an increase in well-being after 4–6 weeks (2nd inquiry, 1st vs. 2nd inquiry *p* = 0.074) and 8–12 weeks [3rd inquiry,1st (23 ± 12) vs. 3rd inquiry (38 ± 20), *p* = 0.009; Figure 1] of telemedical treatment, respectively. In contrast, well-being scores of ADHD patients plateaued around 40% in all three inquiries (1st inquiry 37 ± 21, 2nd inquiry 41 ± 21, 3rd inquiry 40 ± 22), indicative of poor well-being and revealing almost no beneficial effect of the telemedical intervention. Comparing sociodemographic data of both participant groups revealed a similar distribution of both sexes and age (Table 1). No significant difference between groups was also reported for marital status, living situation, education and professional training. However, we found a significantly different distribution of specifications regarding children, labor and financial situation (comparing the distribution of the different categories using Chi-squared test; Table 1): More participants with ADHD lived together with children (17% vs. 4% for participants with depression), while depressive participants were more likely to be unemployed (21%) or disabled (21%) or had financial problems (31% of participants with depression vs. 11% of participants with ADHD with financial debt). As the sub-cohort with depression consisted of significantly more female subjects (Table 1), we also performed a sex-specific analysis searching for sex-specific sociodemographic characteristics in the patient cohorts either diagnosed with depression or ADHD (Table 2). While in the sub-cohort with depression most male subjects were single (85%), the majority of male subjects diagnosed with ADHD lived together with a partner (65%; Table 2). More than a third (37%) of females participating in our study and diagnosed with ADHD had children, while this was not the case for females diagnosed with depression (only 1 female participant with depression was found to have children; Table 2). However, more females with depression also reported a precarious financial situation (Table 2). For all other sociodemographic data assessed no sex-specific differences were found.

**Table 2:** Table depicting sex-related differences of sociodemographic characteristics of participants, either diagnosed with depression or ADHD.

	Depression	ADHD	
	N (%)	N (%)	*p*
Marital status			
Female			0.138
Single	10 (30%)	15 (48%)	
Living with a partner	23 (70%)	16 (52%)	
Missing responses	6	5	
Male			**0.003**
Single	11 (85%)	9 (35%)	
Living with a partner	2 (15%)	17 (65%)	
Missing responses	2	5	
Living situation			
Female			0.786
Living alone	11 (48%)	9 (43%)	
Living with family or friends	6 (26%)	8 (38%)	
Living with a partner	4 (17%)	2 (10%)	
Living in supervised accommodation	2 (9%)	2 (10%)	
Missing responses	16	15	
Male			0.213
Living alone	9 (82%)	9 (50%)	
Living with family or friends	2 (18%)	8 (44%)	
Living with a partner	0 (0%)	1 (6%)	
Living in supervised accommodation	0 (0%)	0 (0%)	
Missing responses	4	13	
Education			
Female			0.592
No completed education	2 (7%)	0 (0%)	
9 years of school education completed	6 (21%)	4 (14%)	
10 years of school education completed	7 (25%)	8 (29%)	
>12 years of school education completed	12 (43%)	15 (54%)	
Not specified	1 (4%)	1 (4%)	
Missing responses	11	8	
Male			0.206
No completed education	0 (0%)	0 (0%)	
9 years of school education completed	0 (0%)	6 (22%)	
10 years of school education completed	5 (62%)	9 (33%)	
>12 years of school education completed	3 (38%)	9 (33%)	
Not specified	0 (0%)	6 (22%)	
Missing responses	7	4	
Professional training			
Female			0.286
Completed apprenticeship	15 (62%)	12 (50%)	
Completed academic studies	2 (8%)	7 (29%)	
No completed professional training	4 (17%)	2 (8%)	
Academic studies on-going	3 (12%)	3 (12%)	
Missing responses	15	12	
Male			0.083
Completed apprenticeship	9 (100%)	13 (52%)	
Completed academic studies	0 (0%)	5 (20%)	
No completed professional training	0 (0%)	4 (16%)	
Academic studies on-going	0 (0%)	3 (12%)	
Missing responses	6	6	
Children			
Female			**0.033**
Children	1 (3%)	8 (23%)	
No children	13 (37%)	13 (37%)	
Children not specified	21 (60%)	14 (40%)	
Missing responses	4	1	
Male			0.576
Children	1 (8%)	3 (10%)	
No children	5 (38%)	16 (53%)	
Children not specified	7 (54%)	11 (37%)	
Missing responses	2	1	
Labor situation			
Female			0.076
Unemployed	6 (17%)	3 (9%)	
Employed	9 (26%)	17 (49%)	
Disables	5 (14%)	0 (0%)	
Retired	1 (3%)	1 (3%)	
Labor situation not specified	14 (40%)	14 (40%)	
Missing responses	4	1	
Male			0.055
Unemployed	4 (31%)	6 (20%)	
Employed	2 (15%)	13 (43%)	
Disables	5 (38%)	2 (7%)	
Retired	0 (0%)	13 (43%)	
Labor situation not specified	2 (15%)	7 (23%)	
Missing responses	2	1	
Financial situation			
Female			**0.049**
Debts	11 (31%)	3 (9%)	
No debts	8 (23%)	13 (37%)	
Financial situation not specified	16 (46%)	19 (54%)	
Missing responses	4	1	
Male			0.236
Debts	4 (31%)	4 (14%)	
No debts	2 (15%)	11 (38%)	
Financial situation not specified	7 (54%)	14 (48%)	
Missing responses	2	2	

Marital status, children, living situation, education, professional training, labor and financial situation were assessed. N = number of participants for whom information was found in medical records. Percentages were calculated as (N/all N responded for a distinct category)*100. A Chi-squared test was used to compare sex-specific differences for the distinct categories and both patient sub-cohorts. Significant differences (*p* < 0.5) between groups are marked in bold.

#### 3.1.2. Clinician-rated psychopathology, mental distress, global functioning, and load of depressive symptoms

Psychopathological symptoms were plausibly distributed according to the two distinct syndromic diagnoses: Significantly more participants with ADHD showed attentional and concentration deficits (Table 3, for attention *p* < 0.001, for concentration *p* = 0.009, Chi-squared test), while the ability to experience joy and the lack of drive was particularly disturbed in depressive patients (Table 3, *p* < 0.001, Chi-squared test). Significantly more depressive participants also reported a history of suicidal attempts (Table 3, *p* < 0.001, Chi-squared test).

**Table 3:** Table depicting psychopathological features drawn from the medical records of study participants at the beginning of telemedical treatment, comparing participants with depression and ADHD.

	Depression		ADHD		
Signs of psychopathology	N	Abnormal	N	Abnormal (%)	*p*
Vigilance	34	3 (9%)	55	0 (0.0%)	0.066
Orientation	34	3 (9%)	55	0 (0.0%)	0.074
Memory	34	6 (18%)	54	14 (26%)	0.489
Perception	34	0 (0.0%)	55	2 (4%)	0.296
Attention	34	9 (26%)	55	36 (65%)	**<0.001**
Concentration	34	18 (53%)	55	41 (75%)	**0.009**
Thought process	34	24 (71%)	54	32 (59%)	0.412
Thought content	34	2 (6%)	55	3 (5%)	0.976
Tricks of the senses	34	1 (3%)	55	0 (0.0%)	0.281
Self-disorder	34	4 (12%)	55	0 (0.0%)	**0.015**
Changes in mood	34	29 (85%)	54	37 (69%)	0.135
Ability to experience joy	34	24 (71%)	55	15 (27%)	**<0.001**
Lack of drive	34	25 (74%)	55	18 (33%)	**<0.001**
Worries, anxiety or fear	34	23 (68%)	55	25 (45%)	0.124
Intrusions	30	2 (7%)	46	2 (4%)	0.553
Compulsive behavior	33	2 (6%)	55	4 (7%)	0.965
Psychomotor function	34	7 (21%)	54	15 (28%)	0.683
Changes in eating habits	34	7 (21%)	55	5 (9%)	0.279
Sleep	34	22 (65%)	55	28 (51%)	0.332
Libido	34	11 (32%)	55	9 (16%)	0.152
Social interaction	34	3 (9%)	55	2 (4%)	0.171
Self-harming behavior	34	2 (6%)	55	3 (5%)	0.932
History of suicidal attempts	45	10 (22%)	61	0 (0.0%)	**<0.001**

N = number of participants for whom information was found in the medical records. The percentage of participants with abnormal psychopathological features was calculated as (N/all N responded for a distinct category)*100 and presented as N(%) with N being the number of subjects with abnormal features for a distinct category. Results for the different psychopathological features among participants with depression or ADHD were compared using Chi-squared tests. *p* < 0.05 was set to be significant. Significant differences between groups for a specific category are marked in bold.

Participants suffering from depression were also more severely affected in the majority of symptoms assessed by the SCL-90R compared to participants with ADHD at baseline (Table 4): At the beginning of the study, depressive participants scored with a value >60 in all nine symptom categories, indicating clinically relevant global mental distress (Table 4). In contrast, mean values for five out of nine sub-categories and all global scores were significantly lower in ADHD patients (all values of *p* < 0.05, Mann–Whitney-U-Test) at baseline. Except for one category (somatization) ADHD patients also crossed the mean threshold of 60, suggesting a relevant burden of psychiatric symptoms in this sub-cohort as well. Matching with patients’ self-reports, clinician-rated scales of global impairment (GAF and CGI) were also significantly different between participants with depression and ADHD (Table 5, *p* < 0.002 for both scales, Mann–Whitney-Test). Depressive participants were clinically more severely affected by mental distress (Table 5). In addition, participants with depression scored significantly higher on the BDI-II (Table 5, *p* = 0.01, Mann–Whitney-Test).

**Table 4:** Detailed initial results of the SCL90-R before the beginning of psychiatric treatment via telemedicine, comparing results of participants diagnosed with depression and ADHD.

	Depression			ADHD			
	N	T Value ≥ 60 (N/%)	Mean ± SD	N	T Value ≥ 60 (N/%)	Mean ± SD	*p*
GSI	52	49 (94.2)	72 ± 7.8	66	50 (75.8)	66 ± 8.4	**<0.001**
PST	52	41 (78.8)	66 ± 9.2	66	42 (63.6)	62 ± 9.1	**0.010**
PSDI	52	50 (96.2)	69 ± 6.1	66	51 (77.3)	65 ± 7.4	**0.001**
Somatization	50	40 (76.9)	66 ± 8.1	63	27 (42.9)	57 ± 11	**<0.001**
Obsessive-Compulsive	50	46 (92.0)	72 ± 7.2	62	56 (90.3)	70 ± 7.9	0.224
Interpersonal Sensitivity	49	42 (85.7)	69 ± 9.2	63	41 (65.1)	64 ± 10	**0.015**
Depression	43	43 (100.0)	74 ± 5.8	58	45 (77.6)	66 ± 9.2	**<0.001**
Anxiety	50	41 (82.0)	70 ± 9.1	63	39 (61.9)	66 ± 10	**0.002**
Hostility	49	38 (77.6)	66 ± 9.4	62	37 (59.7)	63 ± 11	0.156
Phobic Anxiety	50	33 (66.0)	65 ± 11	64	35 (54.7)	60 ± 11	0.072
Paranoid Ideation	48	32 (66.7)	64 ± 10	63	31 (49.2)	61 ± 9.6	0.061
Psychoticism	50	43 (86.0)	67 ± 8.1	64	44 (68.8)	62 ± 8.7	**0.001**

The table provides the results of the three major SCL-90-R indices of distress (GSI, PDSI and PST) as well as the subscales of the nine psychopathological features including the number (N) of participants, the number of subjects with t-values above 60 for the respective subcategory and their percentage relative to all participants (N/all N responded for a distinct category)*100. We also compared the means between both patient groups, using Mann–Whitney-U-Test, revealing significantly higher scores for almost all categories in participants with depression. *p* < 0.05 was set to be significant. Significant differences between groups for a specific category are marked in bold. SD = standard deviation.

**Table 5:** Table revealing the frequency of psychiatric counseling 12 months before the pandemic and during the course of the study during the COVID-19 pandemic, indicating the number of face to face and telemedical counseling sessions.

	Depression		ADHD		
Out-patient treatment (Mean # of doctoral appointments)	N (%)	M (SD)	N (%)	M (SD)	*p*
Within 12 months before the pandemic	4 (7.4)	9 (± 10.4)	3 (4.5)	1.7 (± 1.2)	0.3429
During the pandemic	51 (94.4)	6.4 (± 8.0)	65 (97.0)	3.9 (± 3.4)	0.1830
Via telemedicine	51 (94.4)	4.2 (± 5.0)	65 (97.0)	3.2 (± 2.2)	0.8783
GAF	16 (30.0)	50.6 (± 9.5)	25 (37.3)	62.6 (± 9.7)	**<0.0001**
CGI	24 (44.4)	5.4 (± 0.7)	33 (49.3)	4.7 (± 0.8)	**0.0002**
BDI	19 (35.2)	29.9 (± 16.7)	23 (34.3)	17.8 (± 10.2)	**0.0105**

N = number of participants for whom information was found in the medical records. The table is also depicting the mean scores of the GAF, CGI and BDI-II at the beginning of telemedical treatment for patients diagnosed with depression vs. ADHD. Percentages were calculated as [N/total N of depressive patients (N = 54) or ADHD patients (N = 67)]*100. M = mean, SD = standard deviation. The Mann–Whitney-U-Test was used to assess statistically significant differences between both diagnostic groups. *p* < 0.05 was set to be significant. Significant differences between groups for a specific category are marked in bold.

#### 3.1.3. Utilization of mental health services and satisfaction with telemedical treatment

Participants with depression received on average 6.4 ± 8.0 outpatient doctoral appointments (Table 5). 4.2 ± 5.0 of which were telemedical interventions, while participants with ADHD participated in less doctoral appointments (3.9 ± 3.4) of which the majority where telemedical treatments (3.2 ± 2.2; Table 5). However, this difference was not statistically significant (*p* > 0.05, Mann–Whitney-U-Test). Despite their different treatment outcomes in well-being (Figure 1), both patient cohorts were satisfied with the telemedical interventions they received (77.7% of participants with depression vs. 75.0% of participants with ADHD; Table 6). Both groups evaluated telemedical treatment to be as effective as face to face therapy (51.7% of participants with depression vs. 52.3% of participants with ADHD) and were willing to engage in telemedical treatment in the future again (48.4% of participants with depression vs. 58.6% of participants with ADHD; Table 6). A sex-specific difference regarding outcomes on the WHO-5, BDI-II scores and satisfaction with telemedical treatment could not be identified for both sub-groups.

**Table 6:** Results of the evaluation of telemedical psychiatric counseling by study participants, either diagnosed with depression or ADHD.

	Depression	ADHD	
Telemedical treatment	N (%)	N (%)	*p*
Via phone	30 (93.7)	43 (95.6)	0.675
Via video chat	0 (0)	0 (0)	
Via phone and video chat	2 (6.3)	2 (4.4)	
Technical problems during the telemedical treatment			
Yes	4 (12.9)	6 (13.3)	0.435
No	27 (87.1)	39 (86.6)	
Satisfaction with telemedical treatment			
Strongly disagree	0 (0)	0 (0)	0.692
Disagree	0 (0)	2 (4.5)	
Undecided	6 (22.2)	9 (20.5)	
Agree	8 (29.6)	11 (25.0)	
Strongly agree	13 (48.1)	22 (50.0)	
Telemedical treatment was experienced as effective as therapy in person			
Strongly disagree	2 (6.5)	4 (9.1)	0.979
Disagree	8 (25.8)	9 (20.4)	
Undecided	5 (16.1)	8 (18.2)	
Agree	6 (19.4)	7 (15.9)	
Strongly agree	10 (32.3)	16 (36.4)	
Patients will consider telemedical treatment in the future again			
Strongly disagree	5 (16.1)	8 (18.6)	0.806
Disagree	5 (16.1)	6 (14.0)	
Undecided	6 (19.4)	5 (11.6)	
Agree	4 (12.9)	7 (16.3)	
Strongly agree	11 (35.5)	17 (39.5)	
COVID-19 pandemic influenced mental well-being?			
Agree	18 (60.0)	23 (51.1)	0.637
Disagree	12 (40.0)	22 (48.8)	
How deeply were patients mentally affected by the COVID-19 pandemic?			
Not at all	1 (5.6)	0 (0)	0.322
Slightly	1 (5.6)	2 (8.7)	
Moderate	3 (16.6)	8 (34.8)	
Strong	10 (55.6)	9 (39.1)	
Very strong	3 (16.6)	4 (17.4)	

Feedback considering the mode of telemedical treatment, problems that emerged during telemedical treatment, overall satisfaction and willingness to use telemedical treatment options in the future were evaluated. Participants were also asked to score how deeply their mental health was influenced by the COVID-19 pandemic. N = number of participants that provided feedback at the end of telemedical treatment. Percentages were calculated as (N/all N responded for a distinct category)*100. The Mann–Whitney-U-Test was used to assess statistically significant differences between both diagnostic groups for the different categories, but no significant differences (*p* < 0.05) were found.

### 3.2. Determinants of treatment outcomes among patients with attention-deficit/hyperactivity disorder

#### 3.2.1. Psychopathology, sociodemographic data, psychological burden, global functioning, well-being, and service utilization

Despite their clinically worse condition at baseline, participants with depression reported a significantly improved well-being during the course of the study. No such effect could be demonstrated for participants with ADHD on a group level. Thus, differences in sociodemographic data, signs of psychopathology, SCL-90-R scores, and overall satisfaction with telemedical treatment were investigated between patients with ADHD, that reported improved well-being during telemedical treatment, and participants diagnosed with ADHD without significant improvement on the WHO-5 (Figure 2A). No significant differences among the two subgroups were found for sociodemographic data (Supplementary Table 1), signs of psychopathology (Supplementary Table 2), the SCL-90-R sub-scales and global scores (Supplementary Table 3), and the level of satisfaction with the telemedical intervention (Supplementary Table 4). Clinicians’ ratings of the overall clinical severity of psychiatric symptoms and the ability to participate in daily life using the GAF and CGI also showed no significant difference between both groups at baseline (CGI mean: 5 ± 0 for the group without improvement, mean: 5 ± 0.43 for the group with improvement; GAF mean: 61 ± 0 for the group without improvement, mean: 60.2 ± 10.64 for the group with improvement). Participants diagnosed with ADHD either with or without improvement on the WHO-5 index received an equal amount of telemedical interventions (3.1 ± 1.88 sessions for participants with improvement and 3.4 ± 2.1 sessions for participants without improvement).

#### 3.2.2. Depressive symptoms, sex and familial status

Thirty-three patients with ADHD (49.3% of the participants diagnosed with ADHD) also completed the BDI-II. Participants with ADHD and no improvement or even a further decline on the WHO-5 during the course of the study (Figure 2A), had significantly higher BDI-II scores than participants with ADHD with a more favorable outcome (Figure 2B, *p* = 0.03, Mann–Whitney-U-Test). Thus, predominantly ADHD patients reporting an elevated burden of depressive symptoms were not likely to profit from the telemedical intervention. Furthermore, a sex specific analysis of sociodemographic data revealed that the number of female subjects living with family was higher in the group of patients with a less favorable outcome in the WHO-5 (Table 7). Female sex in general was correlated with the worst outcome in the group of ADHD patients with no improvement on the WHO-5 during the course of the study [Table 7, *r* = −0.675, *p* (two-tailed) = 0.001, Spearman correlation]. In contrast, ADHD patients without children benefited more from telemedical treatment during the COVID-19 pandemic than ADHD patients who were living together with children during the study period [*r* = 0.466, *p* (two-tailed) = 0.02, Spearman correlation]. Further analyses, examining correlations between the outcome on the WHO-5 and different sociodemographic factors (e.g., age, marital status, education, professional training, labor and financial situation) were not conclusive (Table 8).

**Table 7:** Table depicting sex-related differences of sociodemographic characteristics of participants diagnosed with ADHD and with or without an improvement in the WHO-5.

	ADHD		
	No improvement	Improvement	
	N (%)	N (%)	*p*
Marital status			
Female			0.696
Single	5 (50%)	5 (42%)	
Living with a partner	5 (50%)	7 (58%)	
Missing responses	0	3	
Male			0.893
Single	2 (25%)	2 (22%)	
Living with a partner	6 (75%)	7 (78%)	
Missing responses	2	2	
Living situation			
Female			**0.04**
Living alone	2 (25%)	5 (62%)	
Living with family or friends	5 (62%)	0 (0%)	
Living with a partner	1 (12%)	1 (12%)	
Living in supervised accommodation	0 (0%)	2 (25%)	
Missing responses	2	7	
Male			0.368
Living alone	2 (50%)	4 (57%)	
Living with family or friends	1 (25%)	3 (43%)	
Living with a partner	1 (25%)	0 (0%)	
Living in supervised accommodation	0 (0%)	0 (0%)	
Missing responses	6	4	
Education			
Female			0.825
No completed education	0 (0%)	0 (0%)	
9 years of school education completed	1 (11%)	1 (9%)	
10 years of school education completed	3 (33%)	3 (27%)	
>12 years of school education completed	5 (56%)	6 (55%)	
Not specified	0 (0%)	1 (9%)	
Missing responses	1	4	
Male			0.062
No completed education	0 (0%)	0 (0%)	
9 years of school education completed	0 (0%)	5 (50%)	
10 years of school education completed	3 (38%)	2 (20%)	
>12 years of school education completed	5 (62%)	3 (30%)	
Not specified	0 (0%)	0 (0%)	
Missing responses	2	1	
Professional training			
Female			0.856
Completed apprenticeship	4 (50%)	5 (63%)	
Completed academic studies	3 (38%)	2 (25%)	
No completed professional training	0 (0%)	0 (0%)	
Academic studies on-going	1 (12%)	1 (12%)	
Missing responses	2	7	
Male			0.094
Completed apprenticeship	4 (44%)	6 (67%)	
Completed academic studies	4 (44%)	0 (0%)	
No completed professional training	0 (0%)	2 (22%)	
Academic studies on-going	1 (11%)	1 (11%)	
Missing responses	1	2	
Children			
Female			0.098
Children	2 (20%)	1 (7%)	
No children	6 (60%)	4 (29%)	
Children not specified	2 (20%)	9 (64%)	
Missing responses	0	1	
Male			0.403
Children	2 (20%)	1 (10%)	
No children	4 (40%)	7 (70%)	
Children not specified	4 (40%)	2 (20%)	
Missing responses	0	1	
Labor situation			
Female			0.356
Unemployed	1 (10%)	0 (%)	
Employed	6 (60%)	7 (50%)	
Disables	0 (%)	0 (%)	
Retired	0 (%)	0 (%)	
Labor situation not specified	3 (30%)	7 (50%)	
Missing responses	0	1	
Male			0.566
Unemployed	2 (20%)	2 (20%)	
Employed	2 (20%)	5 (50%)	
Disables	1 (10%)	0 (%)	
Retired	1 (10%)	1 (10%)	
Labor situation not specified	4 (40%)	2 (20%)	
Missing responses	0	1	
Financial situation			
Female			0.883
Debts	1 (10%)	1 (7%)	
No debts	5 (50%)	6 (43%)	
Financial situation not specified	4 (40%)	7 (50%)	
Missing responses	0	1	
Male			0.472
Debts	1 (10%)	3 (30%)	
No debts	5 (50%)	3 (30%)	
Financial situation not specified	4 (40%)	4 (40%)	
Missing responses	0	1	

Marital status, children, living situation, education, professional training, labor and financial situation were assessed. N = number of participants for whom information was found in medical records. Percentages were calculated as (N/all N responded for a distinct category)*100. A Chi-squared test was used to compare sex-specific differences for the distinct categories and both patient sub-cohorts. Significant differences (*p* < 0.5) between groups are marked in bold.

**Table 8:** Table revealing the results of the correlation analysis (Spearman correlation) between different sociodemographic characteristics and the results of the WHO-5 inquires (1–3) in patients diagnosed with ADHD, with or without improvement in the WHO-5.

	ADHD			
	Improvement in WHO-5		No improvement in WHO-5	
	Correlation coefficient *r*	*p*	Correlation coefficient *r*	*p*
Sex	0,05754	0,7,801	−0,675	**0.001**
Age	0,06731	0,7,439	0,2098	0,3,746
Marital status	0,3,447	0,126	−0,2,164	0,3,885
Living situation	−0,2,664	0,3,352	−0,2,727	0,3,852
Education	0,1816	0,4,308	0,1,581	0,5,381
Professional training	0,1,158	0,6,173	−0,2,444	0,3,283
Children	0,4,655	**0,0219**	−0,1,648	0,4,875
Labor situation	−0,05997	0,7,807	0,1,025	0,6,672
Financial situation	−0,01829	0,9,324	0,1,223	0,6,076

For both sub-groups absolute differences between results of the 2nd and 3rd WHO-5 inquiry were compared to the results of the 1st inquiry and correlated to sex, age, marital status, living situation, education, professional training, children, labor and financial situation. Provided are the correlation coefficients r for each category and the corresponding *p* values. The level of significance was set to *p* < 0.5.

## 4. Discussion

The data presented here builds on an earlier exploratory study that aimed at describing the changes of symptoms of psychiatric outpatients during telemedical treatment during the COVID-19 pandemic, identifying patient groups with beneficial or less favorable treatment outcomes, determining sociodemographic factors with an impact on the effectiveness of telepsychiatric treatment, and specifying patients’ experiences with telepsychiatric consultations compared to conventional face to face treatment by mental health experts. The objective of the current analysis was to identify factors that distinguish patients with a depressive disorder, who experienced the best treatment outcome, from patients with poorer treatment results on a group level, namely patients with ADHD. Secondly, the data was screened for factors that allowed for a better differentiation between ADHD patients with a satisfactory treatment outcome and ADHD patients with a stagnating or even worsening mental health status.

Comparing the two groups of patients that experienced the highest and the least improvement in well-being during telemedical psychiatric treatment in our study, namely patients with either clinically confirmed depression or ADHD, patients with depression more frequently reported financial debt and unemployment. At the same time patients with ADHD were more likely to have children. Nevertheless, sociodemographic characteristics could not explain the significant difference in treatment outcomes between both groups. Clinical diagnoses of depression and ADHD were mirrored by clinicians’ standardized ratings of psychopathology, substantiating the validity of diagnostic procedures during telemedical treatment. Patients with depression reported a significantly greater impairment in well-being on the WHO-5 index and a higher burden of psychopathology on the SCL-90-R in combination with a lower clinician-rated global functioning on the GAF and CGI scales at baseline. However, during the course of the study, patients with depression experienced a substantial improvement of well-being, while ADHD patients’ well-being scores stagnated or even deteriorated on a group level. There was a statistically non-significant tendency of patients with ADHD to engage in less telemedical appointments than their depressed counterparts. However, patients with depression and participants with ADHD were equally satisfied with the treatment they received. Stratification of patients with ADHD according to the development of well-being during the study period revealed no differences in well-being, psychopathology and global functioning at baseline between patients with an improvement in well-being and those without. Hence, patients with ADHD who experienced improvement during telemedical treatment were not already less impaired at the beginning of treatment. The frequency of telemedical consultations was also evenly distributed between both groups of patients. Yet, ADHD patients with a lack of improvement or even a further decline of well-being during the study reported a higher load of depressive symptoms on the BDI-II than ADHD patients with a favorable treatment outcome. Furthermore, female patients with adult ADHD and patients living with children were more likely to experience an unfavorable treatment outcome.

Generally, quality of life is significantly reduced in patients with ADHD compared to their healthy peers (40). Depressive symptoms and traumatic childhood experiences seem to be important mediators of impairments of well-being among individuals with ADHD. Quality of life can be sustainably improved by evidence-based treatments in adult patients with ADHD, especially after early diagnosis (41). Correspondingly, in a recent, relatively small randomized controlled trial, a combination treatment with CBT and medication was superior in improving quality of life in adult patients with ADHD (42). However, another study found the negative impact of ADHD on quality of life was not significantly reduced in the presence of psychosocial treatment and/or medication among college students with ADHD (43). Whether quality of life can sustainably be enhanced by evidence based treatments in patients with ADHD over their entire lifespan is still an open question. In this context, it has to be noted, that ADHD patients referred to CIMH outpatient services are usually consistently offered evidence based treatments like psychoeducation, stimulant and non-stimulant medication, and/or cognitive behavioral therapy (CBT). Although ADHD is a chronic condition, these treatments reliably alleviate core symptoms of ADHD and improve patients’ psychosocial functioning in the short term. Therefore, despite the chronic nature of ADHD, a stagnation or further decline of well-being among patients with ADHD on a group level in our study was somewhat unexpected. Roughly, one third of the ADHD patients in our sample had a comorbid depressive disorder. This might have had a considerable, detrimental impact on the recovery of these ADHD patients, as ADHD patients without improvement reported a higher burden of depressive symptoms on the BDI-II. Moreover, ADHD has been identified as a relevant factor concerning treatment resistance to antidepressants among patients with major depression and comorbid ADHD (44). Our analyses provide evidence that depressive disorders and a high burden of depressive symptoms might be important contributors to resistance to telemedical treatment among adult patients with ADHD. However, this has to be confirmed in larger, prospective trials.

Attention-deficit/hyperactivity disorder research on psychiatric treatment and support during the COVID19 pandemic has largely neglected adults, older adults and females as well as minority groups (12). On the contrary, especially among young adults and racially and ethnically minoritized subpopulations increases in mental-health related emergency department visits were seen during the COVID-19 pandemic (45). Of note, only recently specific needs as well as differences in psychopathology, social functioning, and developmental trajectories of girls and women with ADHD have been recognized more broadly (46).

On a general level, our findings basically corroborate results from an earlier study that found female children and adolescents with a mental illness during the COVID-19 pandemic to be at a higher risk for psychological burden than their mentally healthy peers (47). Yet, in contrast to their adult counterparts in our study, this effect was less pronounced in individuals with ADHD compared to patients with a depressive disorder. This highlights the need for future studies, specifically addressing the needs of adult female patients with ADHD, since they had the worst treatment outcome in our study. Current long-term data suggest that women diagnosed with ADHD during childhood had impairing problems 17 to 20 years later while rates of remission were relatively low (48). This also confirms results of earlier studies (49–51). Moreover, women with ADHD are at an increased risk of accidents and unintentional injuries, with a higher risk for mild incidents and the same pattern of severe incidents throughout the lifespan compared with men affected by ADHD (52). Women with ADHD display dynamics of emotional dysregulation comparable to female patients with borderline personality disorder, featuring similar levels of symptom intensity and psychopathological instability (53). In fact, severe emotional dysregulation characterizes a cluster of adult ADHD patients associated with significant impairments like depressive mood, negative affect, and elevated psychological distress (54). Remarkably, these patients reported a significantly higher global impairment on the SCL-90-R and elevated BDI scores. Lastly, women were overrepresented in this cluster of adult patients with ADHD. Although we did not screen for emotional dysregulation, this is in line with major findings in our own sub-cohort of adult ADHD patients with insufficient response to telemedical treatment. Therefore, it may be speculated that emotional dysregulation could be a crucial transdiagnostic factor, predicting negative treatment outcomes in women with ADHD. However, prospective studies in larger cohorts of patients with neuropsychiatric disorders are needed to validate this hypothesis.

In this context, it has to be emphasized that the presence of depressive symptoms on the BDI-II does not necessarily imply the clinical diagnosis of depression (55). Furthermore, perceived stress could be an important mediator between ADHD symptomatology and the emergence of depressive symptoms on the BDI-II (56). This could partly explain the correlation of negative treatment outcomes with elevated BDI-II scores in the absence of clinically diagnosed depression in adult patients with ADHD under pandemic conditions in our sample. Conversely, we cannot exclude the possibility, that patients diagnosed with a depressive disorder during telemedical treatment also suffered from hitherto undiagnosed ADHD, which could not easily be confirmed according to current guidelines during acute depression. However, the prevalence of ADHD among patients with the main clinical diagnosis of a depressive disorder was very low in our sample. As ADHD has a strong genetic foundation, it is often present throughout multiple generations of a family (57). Thus, it can be hypothesized, that patients with ADHD in our study might have had a higher risk of having children affected by ADHD as well. Conversely, during the COVID-19 pandemic, caring for children with ADHD could have contributed to a deterioration of parental mental health (58). This could partially explain why adult ADHD patients living without children were less susceptible to a negative response to telemedical treatment in our sample. Furthermore, several studies have shown, that in particular women were affected by a heavy overburden of domestic and family care during the COVID-19 pandemic (1). In part, this could also explain the unfavorable outcome for women and other participants of our study living with children, resulting in reduced time resources for self-care and telemedical psychiatric counseling sessions.

Finally, the results of our study overall corroborate earlier findings that remote communication for psychiatric assessment and treatment during the COVID-19 pandemic was found useful, effective, reliable and satisfactory by adult patients with ADHD (59), albeit “less deep” (60). Nevertheless, subjective satisfaction with telemedical treatment did not correspond with successful treatment in our patient cohort.

There are several limitations that have to be taken into account concerning the interpretation of the above mentioned data. Due to the pandemic situation, a randomization of participants to different treatment modalities (face to face vs. telemedicine) was not feasible. Furthermore, treatment choices were purely guided by patients’ preferences and experienced clinicians’ advice and experience. Thus, heterogeneity in treatment modalities during the course of telemedical treatment, e.g., the administration of behavior-therapy oriented and mindfulness-based interventions vs. generic counseling in combination with psychopharmacological treatment, cannot be excluded. Furthermore, women with mental disorders may have higher odds of COVID-19 infection than males with the strongest gender disparity for ADHD (61). Participants in our study were not routinely screened for their history of confirmed COVID-19. Thus, we cannot rule out that female patients with ADHD in our sample were more often exposed to COVID-19 infections and possible sequelae like long COVID-19 (62). As patients had to give informed consent before enrollment in the study, the sample might not be fully representative of a naturalistic patient cohort due to selection effects. The considerable amount of missing values found in our study is also suggestive of selection effects. In this line, it cannot be excluded that patients with the highest burden of symptoms and the lowest level of functioning dropped out of our study at an early stage of treatment, enriching the study population for individuals with less psychopathology and a higher level of functioning. Although the latter does not apply for the ADHD patients of our sample, it may, on the other hand, be speculated that ADHD patients who experienced a rapid improvement stopped treatment and therefore were not available for follow-up surveys. Lastly, the results of our study need to be replicated in larger patient samples, although our findings are currently plausibly extending previous data. With respect to our findings in patients with ADHD, the limited number of cases precludes any definite conclusions concerning the effectiveness of telemedical interventions in this group of patients.

## 5. Conclusion

When assigning adult patients with ADHD to telemedical treatment options in clinical practice, special attention needs to be paid to monitoring the development of symptomatology and treatment outcomes. A change to face to face treatment should be a low-threshold offer despite patients’ subjective satisfaction with telemedical treatment modalities. Women with ADHD, patients living with children and ADHD patients with a high load of depressive symptoms and/or diagnosed with a depressive disorder might not respond adequately to telemedical treatment. Therefore, such treatment options should be considered with caution during the treatment of patients with the aforementioned characteristics.

Future cross-sectional and longitudinal research should focus on sex differences in ADHD symptoms and treatment outcomes and the development of responsive interventions (63), aiming at more individualized treatment plans in terms of a “precision medicine” (64). Furthermore, prospective investigations should examine the effects of telemedical treatment in larger, representative samples of patients. Especially the transdiagnostic evaluation of treatment effects, e.g., comparing participants with Autism Spectrum Disorder (ASD) to individuals with anxiety disorders, could be of particular interest. This could also substantiate our current findings in a still relatively low number of cases.

## Data availability statement

The original contributions presented in the study are included in the article/Supplementary material, further inquiries can be directed to the corresponding author.

## Ethics statement

The studies involving humans were approved by Ethics committee II of the medical faculty Mannheim, University of Heidelberg approved under license no. 2020-562 N. The studies were conducted in accordance with the local legislation and institutional requirements. The participants provided their written informed consent to participate in this study.

## Author contributions

A-SW and PP designed the study. TR, PP, and A-SW acquired the data. AB developed the online survey tool. A-SW and TP analyzed the data. OH, HT, and AM-L advised and discussed the data. PP, TP, and A-SW wrote the manuscript with the help of all authors. All authors contributed to the article and approved the submitted version.

## Footnotes

^1^ https://www.project-redcap.org/
