# Supplementary material for: Female sex and burden of depressive symptoms predict insufficient response to telemedical treatment in adult attention-deficit/hyperactivity disorder: results from a naturalistic patient cohort during the COVID-19 pandemic
Source: Front Psychiatry. 2023 Oct 5;14:1193898. doi: 10.3389/fpsyt.2023.1193898 (PMC10585110; doi:10.3389/fpsyt.2023.1193898)
Supplement: Supplementary file 1 [file Table_1.docx]

**Supplementary Material**

**Supplementary table 1.** Sociodemographic characteristics of participants diagnosed with ADHD, with or without improvement on the WHO-5 well-being index during the course of telemedical treatment. Sex, age, marital status, children, living situation, education, professional training, labor and financial situation were assessed. N= number of participants for whom information was found in the medical records. Percentages were calculated as (N/all N responded for a distinct category)*100. Results are presented in mean ± SD for age; A Chi-squared test was used to compare patients with ADHD, with or without improvement on the WHO-5, for theses characteristics. Significant differences (p<0.5) between groups for a specific category were not found. M=mean, SD= standard deviation.

**Supplementary table 2.** Table depicting psychopathological features which were extracted from the medical records of participants with ADHD with and without improvement on the WHO-5 well-being index during the course of telemedical treatment. N= number of participants for whom information was found in the medical records. Percentages of individuals with abnormal psychopathological features were calculated as (N/all N responded for a distinct category)*100 and presented as N(%) with N being the number of subjects with abnormal features for a distinct category. Results of ADHD patients with and without improvement on the WHO-5 were compared using Chi-squared tests for each sign of psychopathology. Results are presented in mean ± SD; No significant differences were found comparing groups (p< 0.05 was set to be significant).

**Supplementary table 3.** Results of the SCL90-R at baseline, comparing the results of participants with ADHD - with and without improvement on the WHO-5 - during the course of the study: The table provides the results of the three major SCL-90-R indices of distress (GSI, PDSI and PST) as well as the subscales of its 9 psychopathological features including, the number (N) of participants, the number of subjects with t-values above 60 for the respective subcategory and their percentage relative to all participants ((N/all N responded for a distinct category)*100). The means between both sub-cohorts were also compared, using Mann-Whitney-U-Test, revealing no significant difference between the scores of both cohorts (p< 0.05 was set to be significant).

**Supplementary table 4.** Table depicting the evaluation of telemedical psychiatric counseling by study participants with ADHD, with or without improvement on the WHO-5 during the course of telemedical treatment. Participants were asked for their feedback considering the modality of telemedical treatment, problems that emerged during telemedical treatment, overall satisfaction and if they were willing to use telemedical psychiatric treatment in the future. N= number of participants that provided feedback at the end of telemedical treatment. Percentages were calculated as (N/all N responded for a distinct category)*100. The Chi-Square test was used to assess statistically significant differences between frequencies in both diagnostic groups, but no significant differences (p< 0.05) were found.

**Supplementary table 1**

|  | **ADHD** | | | |  |
| --- | --- | --- | --- | --- | --- |
|  | **No improvement** | | **Improvement** | |  |
|  | **N or M (SD)** | **(%)** | **N or M (SD)** | **(%)** | p |
| **Total Number (N) of recruited patients** | 20 |  | 26 |  |  |
| Female | 10 | 50 | 15 | 51.7 | 0.604 |
| Male | 10 | 50 | 9 | 48.3 |  |
|  |  |  |  |  |  |
| **Age (years)** |  |  |  |  |  |
| Females | 39.1 (11.4) |  | 38.1 (11.9) |  | >0.999 |
| Males | 39.2 (9.9) |  | 37.2 (11.8) |  | >0.999 |
|  |  |  |  |  |  |
| **Marital status** | 18 |  | 21 |  |  |
| Single | 7 | 38.9 | 7 | 33 | 0.762 |
| Living with a partner | 11 | 61.1 | 14 | 67 |  |
| Missing responses | 2 |  | 5 |  |  |
|  |  |  |  |  |  |
| **Living situation** | 12 |  | 15 |  |  |
| Living alone | 4 | 33.3 | 9 | 60 | 0.123 |
| Living with family or friends | 6 | 50 | 3 | 20 |  |
| Living with a partner | 2 | 16.7 | 1 | 6.7 |  |
| Living in supervised accomodation | 0 | 0.0 | 2 | 13.3 |  |
| Missing responses | 8 |  | 11 |  |  |
|  |  |  |  |  |  |
| **Education** | 17 |  | 20 |  |  |
| No school graduation | 0 | 0.0 | 0 | 0.0 | 0.227 |
| 9 years of school education completed | 1 | 5.9 | 6 | 30 |  |
| 10 years of school education completed | 6 | 35.3 | 5 | 25 |  |
| >12 years of school education completed | 10 | 58.8 | 8 | 40 |  |
| Education not specified | 0 | 0.0 | 1 | 5 |  |
| Missing responses | 3 |  | 6 |  |  |
|  |  |  |  |  |  |
| **Professional training** | 18 |  | 21 |  |  |
| Completed apprenticeship | 8 | 44.4 | 11 | 52.4 | 0.143 |
| Completed academic studies | 7 | 38.9 | 2 | 9.5 |  |
| No completed professional training | 0 | 0.0 | 2 | 9.5 |  |
| Academic studies on-going | 3 | 16.6 | 6 | 28.6 |  |
| Missing responses | 2 |  | 5 |  |  |
|  |  |  |  |  |  |
| **Children** | 20 |  | 24 |  |  |
| Children | 4 | 20 | 2 | 8.3 | 0.263 |
| No children | 10 | 50 | 11 | 45.8 |  |
| Children not specified | 6 | 30 | 11 | 45.8 |  |
| Missing responses | 0 |  | 2 |  |  |
|  |  |  |  |  |  |
| **Labour situation** | 20 |  | 24 |  |  |
| Unemployed | 3 | 15 | 2 | 8.3 | 0.752 |
| Employed | 8 | 40 | 12 | 50 |  |
| Disables | 1 | 5 | 0 | 0 |  |
| Retired | 1 | 5 | 1 | 4.2 |  |
| Labor situation not specified | 7 | 35 | 9 | 37.5 |  |
| Missing responses | 0 |  | 2 |  |  |
|  |  |  |  |  |  |
| **Financial situation** | 20 |  | 24 |  |  |
| Debts | 2 | 10 | 4 | 16.7 | 0.658 |
| No debts | 10 | 50 | 9 | 37.5 |  |
| Financial situation not specified | 8 | 40 | 11 | 45.8 |  |
| Missing responses | 0 |  | 2 |  |  |

**Supplementary table 2**

|  | **ADHD** | | | |  |
| --- | --- | --- | --- | --- | --- |
|  | **No improvement** | | **Improvement** | |  |
| **Signs of psychopathology** | **N** | **abnormal (%)** | **N** | **abnormal (%)** | p |
| **Vigilance** | 19 | 0 (0.0 %) | 23 | 0 (0.0 %) | 0.211 |
| **Orientation** | 19 | 0 (0.0 %) | 23 | 0 (0.0 %) | 0.211 |
| **Memory** | 19 | 5 (26.3 %) | 23 | 5 (21.7 %) | 0.789 |
| **Perception** | 19 | 1 (5.3 %) | 23 | 0 (0.0 %) | 0.352 |
| **Attention** | 19 | 10 (52.6 %) | 23 | 13 (56.5 %) | 0.691 |
| **Concentration** | 19 | 13 (68.4 %) | 23 | 15 (65.2 %) | 0.491 |
| **Thought process** | 19 | 9 (47.4 %) | 23 | 10 (43.5 %) | 0.758 |
| **Thought content** | 19 | 1 (5.3 %) | 23 | 2 (8.7 %) | 0.491 |
| **Tricks of the senses** | 19 | 0 (0.0 %) | 23 | 0 (0.0 %) | 0.763 |
| **Self-disorder** | 19 | 0 (0.0 %) | 23 | 0 (0.0 %) | 0.447 |
| **Changes in mood** | 19 | 9 (47.4 %) | 23 | 14 (60.7 %) | 0.150 |
| **Ability to experience joy** | 19 | 4 (21.1 %) | 23 | 7 (30.4 5) | 0.687 |
| **Lack of drive** | 19 | 9 (47.4 %) | 23 | 5 (21.7 %) | 0.216 |
| **Worries, Anxiety or Fear** | 19 | 6 (31.6 %) | 23 | 12 (52.2 %) | 0.319 |
| **Intrusions** | 19 | 0 (0.0 %) | 23 | 1 (4.3 %) | 0.707 |
| **Compulsive behavior** | 19 | 3 (15.8 %) | 23 | 1 (4.3 %) | 0.453 |
| **Psychomotor function** | 19 | 6 (31.6 %) | 23 | 2 (8.7 %) | 0.122 |
| **Changes in eating habit** | 19 | 1 (5.3 %) | 23 | 1 (4.3 %) | 0.316 |
| **Sleep** | 19 | 8 (42.1 %) | 23 | 10 (43.5 %) | 0.956 |
| **Libido** | 19 | 2 (10.5 %) | 23 | 5 (21.8 %) | 0.550 |
| **Social interaction** | 19 | 0 (0.0 %) | 23 | 1 (4.3 %) | 0.592 |
| **Self-harming behavior** | 19 | 1 (5.3 %) | 23 | 1 (4.3 %) | 0.906 |
|  |  |  |  |  |  |
| **History of suicidal attempts** | 19 | 0 (0.0 %) | 23 | 0 (0.0 %) |  |

**Supplementary table 3**

|  | **ADHD** | | | | | |  |
| --- | --- | --- | --- | --- | --- | --- | --- |
|  | **No improvement** | | | **Improvement** | | |  |
|  | **N** | **T- Value ≥ 60 (N/%)** | **Mean** | **N** | **T- Value ≥ 60 (N/%)** | **Mean** | p |
| **GSI** | 20 | 15 (75.0) | 66.1 ± 8.7 | 26 | 22 (84.6) | 66.7 ± 7.2 | 0.894 |
| **PST** | 20 | 12 (60.0) | 60.3 ± 9.6 | 26 | 18 (69.2) | 62.7 ± 8.7 | 0.438 |
| **PSDI** | 20 | 15 (75.0) | 63.8 ± 7.4 | 26 | 20 (76.9) | 65.6 ± 6.9 | 0.477 |
| **Somatization** | 18 | 9 (50%) | 59.6 ± 8.0 | 25 | 10 (40.0) | 57.4 ± 10.1 | 0.579 |
| **Obsessive-Compulsive** | 18 | 17 (94.4) | 70.4 ± 7.6 | 25 | 22 (88.0) | 70.7 ± 7.4 | 0.853 |
| **Interpersonal Sensitivity** | 18 | 7 (38.9) | 61.3 ± 9.9 | 25 | 20 (80.0) | 64.7 ± 10.1 | 0.167 |
| **Depression** | 18 | 15 (83.3) | 65.6 ± 10.0 | 25 | 19 (76.0) | 67.7 ± 8.1 | 0.634 |
| **Anxiety** | 18 | 9 (50.0) | 61.9 ± 10.7 | 25 | 19 (76.0) | 65.7 ± 7.5 | 0.187 |
| **Hostility** | 18 | 10 (55.6) | 62.3 ± 10.5 | 25 | 15 (60.0) | 62.4 ± 10.8 | 0.970 |
| **Phobic Anxiety** | 18 | 11 (61.1) | 60.3 ± 9.0 | 25 | 16 (64.0) | 61.4 ± 10.6 | 0.739 |
| **Paranoid Ideation** | 18 | 7 (38.9) | 58.1 ± 8.7 | 25 | 13 (52.0) | 61.3 ± 10.0 | 0.256 |
| **Psychoticism** | 18 | 13 (72.2) | 62.0 ± 9.7 | 25 | 17 (68.0) | 62.2 ± 7.3 | 0.767 |

**Supplementary table 4**

|  | ADHD | | | |  |
| --- | --- | --- | --- | --- | --- |
|  | No improvement | | Improvement | |  |
| **Telemedical treatment** | **N** | **(%)** | **N** | **(%)** | **p** |
| via phone | 19 | 95 | 21 | 95.5 | 0.945 |
| via video chat | 0 | 0 | 0 | 0 |  |
| via phone and video chat | 1 | 5 | 1 | 4.5 |  |
|  |  |  |  |  |  |
| **Technical problems during the telemedical treatment** |  |  |  |  |  |
| Yes | 3 | 15 | 2 | 10 | 0.555 |
| No | 17 | 85 | 20 | 90 |  |
|  |  |  |  |  |  |
| **Satisfaction with telemedical treatment** |  |  |  |  |  |
| Strongly disagree | 0 | 0 | 0 | 0 | 0.973 |
| Disagree | 1 | 5 | 1 | 4.7 |  |
| Undecided | 5 | 25 | 4 | 19.0 |  |
| Agree | 5 | 25 | 6 | 28.6 |  |
| Strongly agree | 9 | 45 | 10 | 47.1 |  |
|  |  |  |  |  |  |
| **Telemedical treatment was experienced as effective as therapy in person** |  |  |  |  |  |
| Strongly disagree | 2 | 10.5 | 2 | 9.5 | 0.511 |
| Disagree | 6 | 31.6 | 3 | 14.3 |  |
| Undecided | 3 | 15.8 | 5 | 23.8 |  |
| Agree | 4 | 21.1 | 2 | 9.5 |  |
| Strongly agree | 4 | 21.1 | 9 | 42.6 |  |
|  |  |  |  |  |  |
| **Willingness to consider telemedical treatment in the future again** |  |  |  |  |  |
| Strongly disagree | 4 | 20 | 5 | 23.6 | 0.103 |
| Disagree | 2 | 10 | 4 | 19.0 |  |
| Undecided | 4 | 20 | 0 | 0 |  |
| Agree | 5 | 25 | 2 | 9.5 |  |
| Strongly agree | 5 | 25 | 10 | 47.1 |  |
